# The Effect of Processing Methods on Phytochemical Composition in Bergamot Juice

**DOI:** 10.3390/foods8100474

**Published:** 2019-10-11

**Authors:** Domenico Cautela, Filomena Monica Vella, Bruna Laratta

**Affiliations:** 1 Stazione Sperimentale per le Industrie delle Essenze e dei derivati dagli Agrumi (SSEA)—Azienda Speciale della Camera di Commercio di Reggio, via T. Campanella, 12-89125 Reggio Calabria, Italy; dcautela@ssea.it; 2 Consiglio Nazionale delle Ricerche (CNR), Istituto di Ricerca degli Ecosistemi Terrestri (IRET), via P. Castellino, 111-80131 Napoli, Italy; monica.vella@iret.cnr.it

**Keywords:** bergamot juice, juice processing, flavanone glycosides, limonoids, stachydrine, antioxidant

## Abstract

Experimental and epidemiological studies show a positive relation between consumption of citrus juices and reduction of risk for some chronic disorders, such as diabetes and cardiovascular diseases. In particular, the bergamot juice is characterized by noticeable amounts of phytochemicals such as flavanone glycosides, limonoids, and quaternary ammonium compounds, all health-beneficial biomolecules. In vitro and in vivo studies have shown anti-inflammatory, cholesterol-lowering, and anti-diabetic activities attributed to these compounds depending on their chemical structure. However, nutritional content of bergamot juice may vary as consequence of different processing techniques, thus needing to address this claim. For this reason, the objective of this research was to evaluate the effects of different processing systems on the proximate constituents, the composition, and the antioxidant activity of the correspondent juices. Overall, the results indicate that the process employed may influence the chemical composition and the functional properties of the ended juice. Screw press method produced a juice with greater content of flavanone glycosides (ranged from 37 to 402 mg/L) and limonoid aglycones (ranged from 65 to 67 mg/L) than the other processes (*p* < 0.001). However, the process used for extraction of bergamot juice did not affect significantly the *N*,*N*-dimethyl-*L*-proline content (*p* < 0.5). Moreover, the screw press juice showed the highest antioxidant activity with EC_50_ value of 9.35 µg/mL, thus suggesting that this method maintains for health the nutritional quality of a fresh-pressed juice.

## 1. Introduction

Citrus fruits not only contain large amounts of ascorbic acid but are rich sources of bioactive compounds that exert wide varieties of biological functions to human health, including antioxidant, antiinflammation, antimutagenicity, anti-carcinogenicity, and anti-aging [1]. The functional components of citrus fruit include flavanones, limonoids, and quaternary ammonium compounds [2,3,4,5]. Flavonoids and limonoids have shown many pharmacological properties: anticancer, cardiovascular, and anti-inflammatory and antioxidant activity [3,6,7,8]. Recent studies have demonstrated that *N*,*N*-dimethyl-*L*-proline (stachydrine), a quaternary ammonium compound presents in *Citrus* genus fruits in considerable quantities, protects endothelial against the injury induced by anoxiareoxygenation [9] and, more, it ameliorates high-glucose induced endothelial cells senescence through the modulation of the Sirtuin 1 pathway [10].

The fruit of bergamot (*Citrus bergamia Risso et Poit*., Rutaceae) is intensively cultivated in a limited coastal area of Reggio Calabria province (southern Italy), due to both climate and environmental conditions that are favorable for its cultivation. 

The medicinal properties of bergamot were empirically used in the past for traditional uses that include improving cardiovascular function. Nowadays several clinical trials suggest that bergamot derived extract supplementation may produce beneficial effects in subjects with moderate hypercholesterolemia by reducing the plasma lipids and improving the lipoprotein profile [11,12].

Commonly, the bergamot fruit is used mostly for production of cold pressed essential oil (BEo), obtained from the peel by wash-scraping the fruit. BEo is widely employed in perfumery, cosmetic, pharmaceutical, and food industries [13]. Unlike essential oil, which is very appreciate, bergamot juice (BJ) is considered a by-product since it has not found its marketability due to scare information on its composition. The BJ is often used to adulterate other citrus juices that have a wider market like lemon juice, indeed the bitter acid taste, color, and aroma of BJ is very close to lemon juice [14]. 

More recently an increasing interest in BJ marketability arose from its content in bioactive compounds such as flavonoids, limonoids [15,16], and quaternary ammonium compounds [5]. 

Different industrial methods applied for other citrus fruits (oranges and grapefruits) could be employed also to extract the bergamot juice from the endocarp in addition to the hand squeezing method.

The technology used in citrus juice processing is similar throughout the world and operates on whole fruits (such as, FMC In Line system), which extracts the juice and the cold pressed peel oil at the same time or on rasped fruits as the screw press. An overview of citrus processing technologies was described by Cautela ed al [17]. Differences among processing systems and equipment employed in juice production arise both from local traditions and from morphological differences among citrus species.

The quality and phytochemical contents of the juice recovered vary according to juice processing methods [17]. One of the major quality variables of commercial juice is the mechanical pressure used to extract the juice from the fruit. Extraction conditions (hard/soft squeezing) will determine relative levels of juice and peel components. Some of them, such as flavanones, are present in much higher concentration in the peel than in the part considered edible by the consumers [18,19].

The processing techniques at the industrial scale like pasteurization, concentration, and freezing could also modify the initial nutritional and antioxidant content of citrus juices as observed for orange juice processing [20]. 

Some studies report the composition of bergamot juice obtained with manual juicing technologies, while there are limited reports on evaluation of the phytochemical content and compositional characteristics of juices obtained with industrial juicing processes [15,16].

The objectives of this research were to address changes in phytochemicals content and proximate constituents due to differences in the methods of juice extraction on the same batch of bergamot fruit.

Moreover, the antioxidant of the fresh BJ juices, obtained using different juice extraction methods, was to correlate with its phytochemicals content, in order to evaluate the effect of several readily available juicing techniques on the heathy properties of the obtained juices.

## 2. Materials and Methods 

### 2.1. Study Design

**Figure 1:**
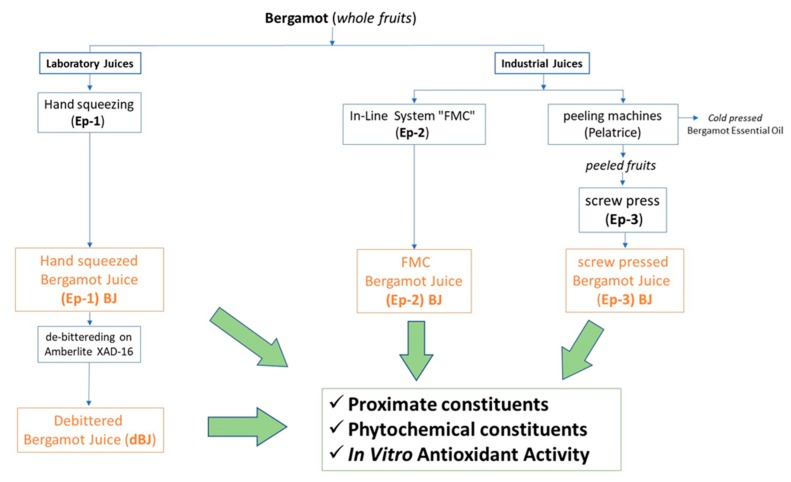
Schematic diagram showing the experimental design applied in this study.

The effect of different processing methods on fresh bergamot juice (BJ) was evaluated by determination of proximate constituents (titratable acidity, total soluble solids, pectins, and free sugars) and by the occurrence of specific phytochemicals (flavanone glycosides, limonoid aglycones, and stachydrine) playing several biological functions in humans. In vitro assay, free radical scavenging activity (RSA) was also determined (Figure 1). 

Fresh bergamot fruits were harvested in January and February 2019 from the experimental field of SSEA agency (Stazione Sperimentale per le Industrie delle Essenze e dei derivati dagli Agrumi, Reggio Calabria, Italy) according to fruits ripening. Once harvested, a random sampling (~10 kg of bergamots) was carried out in order to obtain the batch of fruits juice for laboratory (Extraction process 1: Ep-1). The other harvested fruits (~300 kg) were sent to a local industry that kindly processed the fruits to produce industrial juices using two different extraction systems (FMC in-line extractor (Ep-2) and screw press extractor (Ep-3)).

Laboratory juice (*n* = 3) was prepared by hand squeezing using a manual squeezer (Extraction process 1: Ep-1), filtered through a stainless-steel filter with 1.18 mm (mesh diameter) and placed in 100 mL aliquots in plastic bags, and stored at −20 °C until used. 

Three samples of laboratory juice were debittered by Polystyrene-DVB Resins (Amberlite XAD-16) batch process according to Calvarano et al [21]. The treatment was carried out in a 250 mL beaker containing 25 mL BJ and 5 g of exchange resin, with constant stirring for 20 min at 4 °C. Resin was removed by centrifugation for 10 min at 2000 rpm and at 4 °C.

Industrial juices were obtained using two different extraction systems (Ep-2 and Ep-3):

(a) The Ep-2 system consists of separating the essential oil from the juice in a single operation by using an FMC in-line extractor (FMC Corporation, Fairway Avenue Lakeland, FL-USA.). The FMC squeezer is designed to separate the fruit into three different parts: peel and seeds; peel oil and washing water; and juice. In this extractor, a whole fruit is positioned in one of opposed inter-fitting cup halves which were provided with means for cutting the peel or rind at diametrically opposed areas. Concurrent movement of one or both cups toward each other compresses the fruit and produces the diametrically opposed cuts. Cutting and compressing ruptures the juice sacks and the juice flows through one of the cut areas of the peel, through a screen, and to a collecting trough.
(b) In the Ep-3 system, oil extraction precedes juice extraction. In this case the essential oil is extracted from the whole fruit by rasping, using a machine called “pelatrice.” The rasped fruits are then moved into a juice extractor (POLYCITRUS ZX2, Fratelli Indelicato S.r.l., Catania, Italy). This machine consists of a screw press extractor extracting juice by crushing and pressing whole fruit with a rolling screw. The screw press type operates by pressing the screw horizontally while conveying the pomelo flesh along the perforated cylindrical container. Juices flow out via the perforated cylinder and the wastes are ejected at the end plate.

Detailed descriptions of these processing were described by Cautela et al. [17]. In particular, the industrial juices were sampled during on-line processes, where 1 L of sample was firstly collected at regular intervals and then filtered through a stainless-steel filter (1.18 mm mesh diameter). The fruit juices were placed in 100 mL aliquots in plastic bags and stored at −20 °C until used.

### 2.2. Sample Preparation

Bergamot fruits were harvested and processed within 24 h. Before the preparation of laboratory juice, the fruits (3 batches of ~3 kg) were first washed with water (Milli-Q grade), to remove dust and pollutants from the exocarp, then drained and finally dried on paper. 

For industrial juices, on receival to local industry, bergamot fruits were washed with plain water to remove leaves and dust then drained before processing. A batch of ~150 kg of fruits was employed from FMC In Line juicing extraction. Meanwhile the remaining fruits (1 batch ~150 kg) were rasped employing “pelatrice” machine according to processing line of the industry. The rasped fruits were further washed with water prior to screw press juice extraction in order to reduce the oil content. 

### 2.3. Reagents and Standards 

*N*,*N*-dimethylformamide (anhydrous, 99.8%), 2,2-diphenyl-1-picrylhydrazyl (DPPH), ascorbic acid (≥99.0%) , flavonoids analytical standard with purity ≥97.0% (neoeriocitrin, eriocitrin, narirutin, naringin, hesperidin, neohesperidin), limonoid aglycones analytical standard with purity ≥95.0% (limonin, nomilin), and the HPLC-grade solvents were purchased from Sigma Chemical Co. (St. Louis, MO, USA). *N*,*N*-dimethyl-*L*-proline (≥95.0%) was purchased from Extrasynthese (Genay, France). Analytical-grade water (resistivity ≥18 MΩ cm) and all other solvents and reagents of analytical grade were obtained from Carlo Erba Reagents (Milan, Italy).

### 2.4. Proximate Constituents

The proximate constituents’ determinations were conducted in triplicate on all samples, according to the International Federation of Fruit Juice Producers (IFU) methods [22] of the European Fruit Juice Association (AIJN). The juices were thawed at 4 °C prior to analysis. The analytical determinations were carried out using the following methods: soluble solids, expressed as %*w*/*w* (°Brix), were determined according to IFU-8 method employing a RE20B refractometer (Mettler Toledo SpA, Novate Milanese Italy); total acidity, expressed as citric acid monohydrate (g/L), was obtained by titration against NaOH 0.25 N until pH of 8.1 according to IFU-3 method; ascorbic acid was assessed according to IFU-17 method by titration with 2,6-dichloroindophenol; total and water-soluble pectins were evaluated according to IFU-26 method; sugars (glucose, fructose, and sucrose) were determined spectrophotometrically by enzymatic assay kits (R-Biopharm AG, Darmstadt, Germany) according to IFU-55 and IFU-56 methods.

### 2.5. Analysis of Flavanone Glycosides 

Flavanone glycosides content of the thawed juice were determined according to Cautela et al. [14]. Briefly, 10 mL of thawed juice was shaken with 20 mL of a 1:1 (*v*/*v*) mixture of 0.25 M *N*,*N*-dimethylformamide/ammonium oxalate and 20 mL of analytical-grade water and then filtered on 0.45 μm PTFE Pall filters.

The analyses were performed using a Surveyor autosampler LC system, interfaced with a PDA detector (Thermo Finnigan, Waltham, MA, USA) equipped with Xcalibur 3.1 software (Thermo Fisher Scientific, Waltham, USA).

A volume of 5 μL was employed for the analysis on a Supelco Spherisorb ODS2 HPLC Column (250 × 4.6 mm), and the column was thermostatically controlled at 35 °C. The elution was conducted, as already reported [23], by employing 0.3% acetic acid solution (solvent A) and acetonitrile (solvent B). A gradient elution was performed as the following: the initial solvent was 90% A and 10% B; the gradient elution was changed from 10% to 20% B in a linear mode for 15 min; this composition was maintained at isocratic flow for 10 min; the solvent B reached 50% in 10 min and from 50 to 90% B in 10 min. 

PDA data were recorded in the 200–600 nm range, the identification of the flavanone glycosides was based on retention time and PDA spectra, and quantification was achieved by external standard calibration. Standard calibration curves were prepared in a concentration range 0.001–0.100 mg/mL. The calibration curves with the external standards were obtained using concentration (mg/mL) with respect to the area obtained from the integration of the PDA peaks at a wavelength of 284 nm. The quantification was achieved by comparison with the calibration curve obtained with standard solutions. The reproducibility of the detector response at each concentration level was evaluated by a triplicate injection of standard mix and expressed as percentage of relative standard deviations (RSD%). The RSDs were expected to be less than 2%. The limits of detection (LOD) were established at a signal to noise ratio (S/N) of 3. The limits of quantification (LOQ) were established at a signal to noise ratio (S/N) of 10. LOD and LOQ were experimentally verified by the nine injections of reference compounds in LOQ concentrations.

### 2.6. Determinations of N,N-dimethyl-L-proline (stachydrine) by HPLC-ESI-MS/MS

Stachydrine content was determined according to Servillo et al. [24] without any sample preparation except the centrifuged of the thawed juice diluted 1:25 (*v*/*v*) with 0.1% formic acid. Further HPLC-MS/MS analyses were performed on an Agilent 1100 Series liquid chromatograph equipped with an LC-MSD SL quadrupole ion trap. The separations were executed with a 150 × 3.0 mm i.d., 5 μm, Supelco Discovery-C8 column, at a flow rate of 100 μL/min. The chromatography was conducted isocratically with 0.1% formic acid in water. Samples of 20 μL of standard solutions or diluted juice sample were injected. 

The conditions for ESI-MS/MS analyses, made in positive ion mode, utilizing nitrogen as the nebulizing and drying gas, were nebulizer pressure, 30 psi; drying temperature, 350 °C; and drying gas, 7 L/min. The ion charge control (ICC) was applied with the target set at 10,000 and maximum accumulation time at 20 ms. In order to obtain efficient collision induced fragmentations of the positively charged parent ion, the ion trap, molecular weight cutoff, and amplitude potential and other instrumental parameters were previously optimized for each analyte. The retention time (expressed in min) and peak areas of the monitored fragment ions were determined by the Agilent software Chemstation, version 4.2 (Agilent Technologies Inc., Santa Clara, CA USA). 

The quantification was achieved by comparison with the calibration curve obtained with standard solutions.

### 2.7. Determinations of Limonoid Aglycones in BJ

The determinations of limonoid aglycones in BJ were conducted according to Esposito et al. [25]. Briefly, 10 mL of the juice sample was centrifuged at 2500 g for 10 min and loaded on Millipore C18 Sep Pak cartridge rinsed with 2 mL of methanol and then 5 mL of deionized water before the use. The cartridge was further washed with 20 mL of deionized water and limonoid aglycones were eluted with 50 mL of methanol. The extracts were dried using a rotary evaporator (IKA RV8, IKA®-Werke GmbH & Co. KG, Staufen, Germany) and resuspended in 1 mL of acetonitrile.

The limonoid aglycones were quantitated by RP chromatography using a Surveyor LC pump, a Surveyor autosampler, coupled with a photodiode array detector (PDA (Thermo Finnigan, Waltham, MA, USA), equipped with Xcalibur 3.1 software (Thermo Fisher Scientific, Waltham, MA USA).

Separations were achieved using a C-18 column (Phenomenex Luna 5 μ C18, 250 × 4.60 mm) and monitoring the wavelengths at 250 nm. The mobile phase consisted of a gradient program that began at methanol 10%, acetonitrile 0%, and water 90% and ended at methanol 41%, acetonitrile 10%, and water 49% in 45 min. The flow rate was 1 mL/min and the injection volume was 5 μL. Sample peak identifications were achieved by comparing retention times from the sample peak with those of standards run under identical conditions. Concentrations were determined using the external standard method.

### 2.8. In Vitro Antioxidant Activity

The free radical scavenging activity (RSA) of the bergamot juice was evaluated by 2,2’-diphenyl-1-picrylhydrazyl (DPPH) assay according to the procedure of Blois [26]. Prior of spectrophotometrically measurement, thawed juices were centrifuged at 10,000 rpm in an 5804R Eppendorf centrifuge (Eppendorf srl, Milano, Italy) for 5 min at room temperature and diluted with analytical-grade ultrapure H_2_O with a concentration ranging from 5 to 20 µL/mL. 

Briefly, 150 μL of bergamot diluted juices were mixed with 1.35 mL of 60 μM DPPH methanolic solution. The absorbance reduction at 517 nm of the DPPH was determined continuously for 40 min. The RSA was calculated as a percentage of DPPH discoloration, using the following equation:

(1) $$\%\;\mathrm{RSA}=\;\left[\frac{\left(A_{\mathrm{DPPH}}\;-{\;A}_{s}\right)}{A_{\mathrm{DPPH}}}\right]\times \;100$$

where A_S_ is the absorbance of the solution when the extract was added and A_DPPH_ is the absorbance of the DPPH solution. The extract concentration (EC) necessary to achieve a 50% of radical DPPH inhibition (EC_50_) was obtained by plotting the RSA percentage as a function of juice concentrations and was expressed as µL/mL. 

Ascorbic acid was used as a reference standard (positive control) and dissolved in ultrapure H_2_O to prepare a 100 µg/mL stock solution. The antioxidant activity of standard ascorbic was tested at various concentrations (10, 15, 25, 50, 60 μg/mL).

To standardize DPPH results, the antioxidant activity index (AAI), proposed by Scherer and Godoy [27], was calculated as follows:

AAI = DPPH concentration in reaction mixture/EC_50_. (2)

Samples were classified as showing poor antioxidant activity (AAI < 0.5), moderate (0.5 < AAI < 1.0), strong (1.0 < AAI < 2.0), and very strong (AAI > 2.0) as reported by Scherer and Godoy [27].

### 2.9. Statistical Analysis

Statistical analysis was performed using the XLSTAT software, version 2016 (Addinsoft, Paris, France. All samples were analyzed in triplicates and the results were expressed as mean ± standard deviation (SD) after a normality distribution Kolmogorov–Smirnov test. Means, SD, calibration curves, and linear regression analyses (*R*^2^) were determined using Microsoft Excel 2013 (Microsoft Corporation, Redmond, WA, USA).

Statistical comparisons were carried out by analysis of variance (ANOVA) and post hoc Tukey–Kramer tests. A p value less than 0.05 was considered statistically significant. All tests were two tailed.

The Pearson’s correlation test was performed by using Microsoft Office Excel 2016 software. The correlation coefficients (*r* values, *p* < 0.05) were obtained to reveal the relationships between antioxidant activity index (AAI) and the content of flavanone glycosides, ascorbic acid, *N*,*N*-dimethyl-*L*-proline (ProBet), and limonoid aglycones.

## 3. Results

### 3.1. Differences in the Proximate Constituents Between the Juices Obtained with Different Processing Systems

The use of different juice extraction systems had influence on proximate constituents of bergamot juice and the outcomes of this study are summarized in Table 1. Bergamot juice was very acidic and mainly contained citric acid, which contributes significantly to the composition of this parameter. Acidity, as citric acid monohydrate, ranged from 41 g/L for Ep-3 juice to 43 g/L in Ep-1. The content of total soluble solids expressed as degree Brix (%*w*/*w*) did not exceed 10% in laboratory and industrial juices. Brix values of harsh (EP-3) and soft (EP-1) squeeze juices remained relatively consistent. The average content of free sugars ranged from 9 g/L of glucose and fructose and 17 g/L of sucrose both in laboratory than in commercial squeezing juice.

It was clear that the different methods employed for the extraction of BJ did not affect significantly the quality traits of the juice, as no significant differences were detected in total soluble solids, titratable acidity, and sugar contents. Total soluble solids ranged from 9.0 ± 0.2% to 9.5 ± 0.4% in BJ obtained by FMC (Ep-2) and hand squeezed (Ep-1), respectively. The greatest contribution to the total soluble solids content was due to citric acid and this fact was also the evidence for the highest acidity value showed in Ep-1. 

The average content of L-ascorbic acid in BJ was 415 mg/L and was similar in all juice. The effect of the system of extraction was also unrelated as regards L-ascorbic acid content. 

Although it was interesting to note that harsh squeeze processed juice (EP-3) had considerable higher amount (*p* < 0.005) of pectic fractions (total and water-soluble pectins) than fresh hand squeezed juices (Ep-1) and soft squeeze processed juice (Ep-2) (Table 1).

### 3.2. Phytochemical Content in Bergamot Juices Obtained with Different Juice Extraction Systems

**Figure 2:**
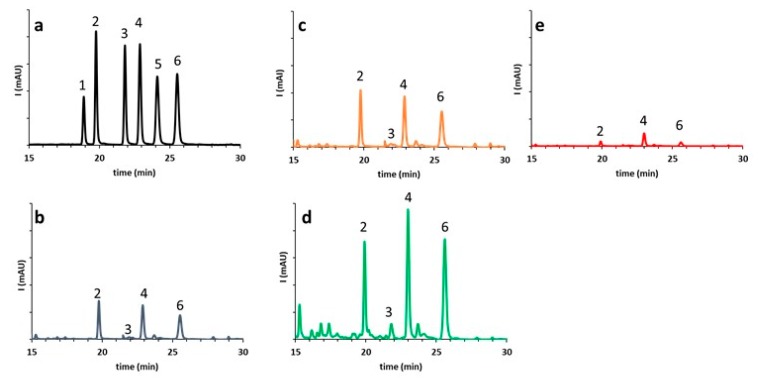
Comparison of RP-HPLC-DAD chromatograms at 284 nm of flavanone glycosides in bergamot juice (BJ) obtained using 3 processing methods: (**a**) Standard mixture of eriocitrin (1), neoeriocitrin (2), narirutin (3), naringin (4), hesperidin (5), neohesperidin (6). (**b**) Hand squeezed BJ (Ep-1); (**c**) FMC–BJ (Ep-2); (**d**) BJ obtained by screw press of peeled fruits (Ep-3); (**e**) debittered bergamot juice (dBJ).

BJ was characterized by noticeable amounts of flavanone-7-O-neohesperidosides (naringin, neoeriocitrin, and neohesperidin) and lower amounts of flavanone-7-O-rutinosides (narirutin). The typical flavanone glycosides pattern of bergamot juice was shown in Figure 2 (panels b–d). The detection limits (LOD) found using RP-HPLC-DAD analysis were 0.5 mg/L, while the calculated limits of quantification (LOQ) were 1 mg/L for each flavanone glycosides. The validation process of HPLC analysis showed a good resolution of all components (Figure 2a), excellent linearity, as confirmed by the correlation coefficient *R*^2^, ranging from 0.990 to 0.998, and a good precision, at the concentration level tested, since the coefficient of variation (CV) values were <5% for all the analytes. Considering the involvement of procedures as extraction, filtration and dilution of sample, the precision and the accuracy of the analytical method were acceptable with intra/inter assay coefficient of variation below 2.3% and 3.9%. 

Results reported in Table 2 and Figure 2 suggested that BJ obtained by screw press (Ep-3) had significantly (*p* < 0.001) higher levels of flavanone glycosides (neoeriocitrin, naringin, neohesperidin) compared to hand squeezed juices (Ep-1) and BJ processed by and FMC (Ep-2). No significant variation in flavanone glycosides content was noticed in the hand squeezed juices and BJ obtained by Ep-2 (*p* < 0.1).

Naringin was the most abundant flavonoid present (394 ± 83; 175 ± 58; and 97 ± 13 mg/L in Ep-3, Ep-2, and Ep-1, respectively). The content of neoeriocitrin ranged from 114 ± 20 mg/L in Ep-1 juice to 402 ± 122 mg/L in Ep-3 juice, while the concentration of neohesperidin was found to be about 1.5- to 1.7-fold lower than naringin content. 

Data from Table 2 showed that *N*,*N*-dimethyl-*L*-proline was the main proline derived osmo-protectant compounds present in BJ. The process used for extraction of BJ did not affect significantly the *N*,*N*-dimethyl-*L*-proline content in juice (*p* < 0. 5).

As reported in Table 2, the content of limonoid aglycones, limonin, and nomilin, increased significantly in the screw press (Ep-3) BJ with an average content of 65 mg/L. BJ obtained by FMC (Ep-2) did not significantly differ from hand squeezed (Ep-1) juice as regarding the limonin content (*p* < 0.05), while nomilin appeared to be lower in the Ep-1 juice.

Since bitterness in BJ was primarily related to two classes of compounds: neohesperidose O-flavanone glycosides (neoeriocitrin, naringin, and neohesperidin) and limonoids (limonin, nomilin), the content of these compound was evaluated in BJ treated with polystyrene-DVB resins (Amberlite XAD-16) batch process. In Table 2 the content of flavanone glycosides and limonoid aglycones of debittered BJ samples was also reported. The effect of resin treatment significantly reduced (*p* < 0.001) the amount of the flavanone glycosides, limonin, and nomilin (Figure 2, Table 2), but seemed to not affect L-ascorbic acid and *N*,*N*-dimethyl-*L*-proline, which did not significatively differ (*p* < 0. 5) from fresh hand squeezed BJ (Table 2).

### 3.3. In Vitro Antioxidant Activity

**Figure 3:**
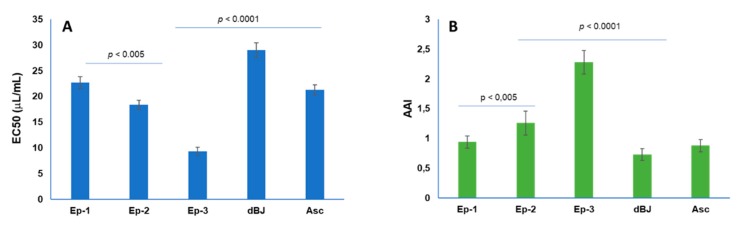
Antioxidant activity expressed as EC_50_ (**A**) and antioxidant activity index (AAI) (**B**) of bergamot juice obtained using 3 different juice extraction methods: hand squeezing (Ep-1); FMC (Ep-2); screw press of peeled fruits (Ep-3); and in debittered bergamot juice (dBJ). Asc: ascorbic acid (positive control). Error bar represent standard deviation (*n* = 3).

The antioxidant activity of the BJ juices obtained using 3 different juice extraction methods were estimated by DPPH radical scavenging assay (Figure 3), ascorbic acid was used as the reference standard. The antioxidant activity of debittered bergamot juice (dBJ) was also evaluated in order to find a possible correlation between the free radical scavenging activity (RSA) of fresh processed BJ and its’ content in phytochemicals.

Ascorbic acid, at a concentration of 20 μg/mL, exhibited a percentage inhibition of 51.1% and a calculated EC_50_ value of 21.25 μg/mL.

After 40 min, screw press (Ep-3) BJ showed the highest DPPH radical scavenging activity, (EC_50_ was 9.35 μg/mL, *p* < 0.0001). The EC_50_ of hand squeezed (Ep-1) BJ was significatively lower than FMC juice (Ep-2) (*p* < 0.005). Debittered bergamot juice (dBJ) showed an EC_50_ value of 29 µg/mL, significantly higher than the other juice (*p* < 0.0001). It is mainly due to the L-ascorbic acid content, as previously reported in Table 2. 

A very strong antioxidant activity (AAI > 2.0) was observed only in screw press (Ep-3) BJ, while the AAI of FMC juice (Ep-2) was significatively higher than Ep-1 (hand squeezed) and debittered bergamot juice (dBJ) (*p* > 0.05). The present results demonstrate stronger activity of EP-3 BJ than that of the standard antioxidant ascorbic acid (*p* > 0.0001).

Moreover, the BJ debittered with exchange resin showed significantly lower (*p* < 0.0001) total antioxidant activity due to minor content of flavanone glycosides and limonoid aglycones (Table 2, Figure 3). The exchange resin process led to a significant decrease (*p* < 0.005) in the total concentration of these compounds compared with samples of fresh juice, while no effects were observed on L- ascorbic acid, which ranged from 387 to 402 mg/L, and *N*,*N*-dimethyl-*L*-proline (Table 2).

**Figure 4:**
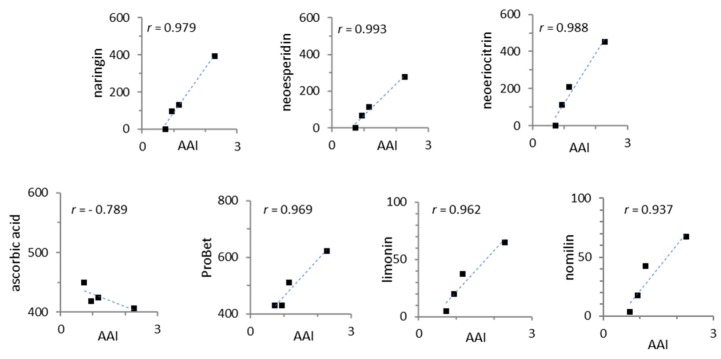
Pearson test correlation coefficients (*r*) among the antioxidant activity index (AAI) and the content (mg/kg) of flavanone glycosides, ascorbic acid, quaternary ammonium compounds, and limonoid aglycones in the BJ (*p* < 0.05).

Correlations between the antioxidant activity index (AAI) and the content of flavanone glycosides, ascorbic acid, *N*,*N*-dimethyl-*L*-proline (ProBet), and limonoid aglycones were calculated using the Pearson test in order to find out if parameters were statistically correlated (Figure 4). Strong positive correlations (*r* > 0.95) were determined for all compounds with the same exception: only a high negative correlation was observed for ascorbic acid content and AAI. 

Results in Figure 3 and Figure 4 indicated that the antioxidant activity index of BJ was mainly due to ascorbic acid content and increased together with the flavanone glycosides and limonoid aglycones content according to processing of juice extraction. 

## 4. Discussion

The objectives of this study were to evaluate the effects of different juice extraction systems on proximate constituents, bioactive components composition, and antioxidant activity of bergamot juice. 

Beneficial activities of the components of citrus juices for human health were primarily ascribed to its antioxidant activity mainly due to L-ascorbic acids and polyphenols, like flavanone glucosides [16]. Despite these beneficial effects, the unprocessed fresh bergamot juice showed sour taste due to a high citric acid content (Table 1) and a bitter taste due to limonoids and flavanone-7-O-neohesperidosides which were also the most dominant bitter principles (Table 2). 

Flavanones usually occurred as O-glycosyl derivatives, with the sugar moiety bound to the aglycone hydroxyl group at either C7 or C3. Among these compounds, the O-diglycosides were a dominant category and their structures were usually characterized by the linkage of either neohesperidose or rutinose to the flavonoid skeleton. The bitterness caused by flavanone-7-O-neohesperidosides was often referred to as ‘primary’ bitterness, while flavanone-7-O-rutinosides were tasteless [28]. 

The amount of the flavanone glycosides depends on the species and of the productive processes used (time and intensity of extraction, line production technology, and quality of the fruit used). It is known that a higher content of flavonoids and limonoids were found in albedo and membranes than in juice sacs [29]. Since there were several methods of extraction juice, each processing method may have its own characteristics in terms of the concentration of bioactive compounds as well as juice quality (proximate constituents).

Hand squeezing (Ep-1) was compared with two industrial squeezing methods: a “soft” juice extraction process (FMC single-strength extraction method, Ep-2) and a “hard” juice extraction process (Ep-3) operated by a screw press of peeled fruits, to evaluate their influences on health components of BJ. 

### 4.1. Proximate and Phytochemical Content in Bergamot Juices Obtained with Different Extraction Systems

As regarding the proximate constituents of Ep-1 BJ (hand squeezed bergamot juice), results were in accordance with the previous report [14]. As showed in Table 1, juice extraction techniques used in this study did not significantly affect same proximate constituents related to juice quality parameters: soluble solids, acidity, L-ascorbic acid, and free sugars. 

The range of variability of total acidity of the samples analyzed, expressed as g/L of monohydrated citric acid, was 37–41 g/L. These values were in good agreement with those previously reported by Calvarano et al [21] and Cautela et al. [14]. As for total acidity content, in this study, significant differences were not observed due to different juice extraction techniques considering both soluble solid and free sugars content (Table 1). If compared to the other citrus juices, the amount of free sugars in bergamot was comparable with that of grapefruit and lemon [14,22].

In citrus juices, pectin was one of the major components of the suspended cloud material that imparts desirable appearance, texture, and flavor [30]. Since the amount of pectins was higher in the albedo and in the membranes, the content of pectins present in the juice depended on the juice extraction system used. Highest values were found in BJ obtained by the “hard extraction process” (Ep-3) compared to the juices obtained by the “soft extraction process”. Results from Table 1 also showed that juice extractor pressure affected the water-soluble and total pectins content that increased about 1.5-folds in Ep-3 BJ.

Based on the contents of L-ascorbic acid found in all samples, BJ was a good source of vitamin C; in fact, a serving of BJ (240 mL) would cover the adult reference daily intake (RDI). 

A glass of BJ also provided approximately 100 mg of stachydrine (*N*,*N*-dimethyl-*L*-proline) making this compound a valid biomarker of orange or other citrus juice consumption [31,32]. Furthermore, *N*,*N*-dimethyl-*L*-proline could contribute to the fine direct/indirect regulation, the endothelial function via the modulation of nitric oxide synthase and cellular senescence pathway [9,10]. 

A randomized, crossover, double-blind, controlled study performed in overweight and obese subjects showed that stachydrine was a useful biomarker to differentiate the intake of orange juice containing different amount of flavanones [32]. Furthermore, the study reported a positive correlation between the consumption of orange juice with a high content of flavanones and the improved relation of oxidative stress and inflammatory biomarkers [32].

The content of flavanone glycosides in hand squeeze (Ep-1) BJ (Table 2) were in good agreement with the data of flavonoid content in bergamot juice reported in literature [14,15,16]. The most abundant flavanone glycosides in bergamot fruits were naringin, neohesperidin, and neoeriocitrin. Among these, naringin was found to lower total cholesterol and low-density lipoprotein cholesterol levels in plasma [33] and has been also evaluated for its probable protective actions on pre-neoplastic lesions [34]. 

A clear technological effect on the flavanone glycosides (Table 2, Figure 2) and limonoid aglycones (Table 2) could be suggested, depending on the juice extraction system used. 

Limonoid aglycones showed numerous pharmacological activities [7] and were the most abundant in seeds and peels [16,25]. This could explain the significatively high level (*p* < 0.05) of limonin and nomilin in EP-3 BJ compared to other juices. 

The increase of flavanone glycosides with increasing extraction pressure were in agreement with Gil-Izquierdo et al. [20], who reported the effect of processing techniques at an industrial scale on orange juice antioxidant and beneficial health compounds. 

### 4.2. In Vitro Antioxidant Activity

In our study the total antioxidant activity of BJ was evaluated using radical scavenging assays based on single electron transfer (SET) mechanisms (2,2-diphenyl-1-picrylhydrazyl, DPPH assays).

The DPPH assay measured the so-called radical-scavenging activity (RSA) which is the ability of extract constituents to scavenge reactive species to stop the initiation or propagation of oxidizing chain reactions [26].

The interaction or synergistic effect among the nutrients and/or bioactive compounds contained in citrus fruits could contribute to their antioxidant activity. Figure 3 illustrated the results of the antioxidant activity obtained for BJ tested in the present study.

Screw press (Ep-3) BJ exhibited the lowest EC_50_ value. Since the ascorbic acid and stachydrine content in all juice was comparable. Furthermore, the high flavanone glycosides, stachydrine, and limonoid aglycones content in screw press (Ep-3) BJ may also contribute to its potent DPPH radical activity. In fact, data reported in Figure 3, confirmed that the debittered bergamot juice (dBJ), had significant higher EC_50_ value. The contribution of antioxidant activity, in dBJ, was due to the ascorbic acid and stachydrine content since, in batch operations, cross-linked divinyl benzene-styrene resin reduced the flavanone glycosides and limonoid aglycones up to 90% (Table 2). The debittering processing with these resins had no effect on the minerals, acid, and amino acids content of the juice, as reported by Kimbal for navel orange juice [35]. 

In the present study, the contribution of ascorbic acid to the antioxidant activities of BJ was found to be moderate (Figure 4). Miller and Rice-Evans [36] had underlined the significant contributory role of polyphenols in the total antioxidant activity of long-life orange juice even if ascorbic acid was present at a higher concentration (Table 1). 

## 5. Conclusions

BJ can be a good dietary source of nutrients like L-ascorbic acid and phytochemicals with antioxidant and health properties. However, nutritional content of bergamot juice varied as consequence of different processing techniques. Our results indicated that the juice extraction processes employed could influence the chemical composition and functional properties of BJ. The industrial processed juice, obtained by conditions (Ep-3), was markedly different from the fresh squeezed juice in the same proximate constituents as pectic substances and phytochemicals content. 

Results from this study suggest that extracting juice under harsh conditions (Ep-3) increased the amount of phytochemical content and total antioxidant activity than FMC and hand squeezing juicing process. 

Several limitations of the present study must be considered. This research looked at the effect of juice processing on the phytochemical content of the resulting juice. Further studies are necessary to understand the influence of the processing conditions (thermally or non-thermally) on increased shelf life of BJ. 

Despite these beneficial effects, the unprocessed fresh bergamot juice showed a bitter taste due to limonoids and flavanone-7-O-neohesperidosides, hence a sensory evaluation should be addressed in order to evaluate consumer acceptance of processed BJ. 

As the health benefits from the phytonutrients was better understood by addressing their bioavailability, future efforts will therefore aim to compare the different types of juices considering the bioavailability of phytonutrients in clinical trials.

## Figures and Tables

**Table 1:** Proximate constituents (mean ± standard deviation, *n* = 3) of bergamot juice obtained using 3 different extraction methods: hand squeezing (Ep-1); FMC (Ep-2); screw press of peeled fruits (Ep-3).

	Ep-1	Ep-2	Ep-3
Total soluble solids (%)	9.5 ± 0.4 ^a^	9.0 ± 0.2 ^a^	9.1 ± 1.2 ^a^
Acidity * (g/L)	43.0 ± 3.0 ^a^	42.5 ± 3.5 ^a^	41.0 ± 3.9 ^a^
L-ascorbic acid (mg/L)	421 ± 25 ^a^	415 ± 30 ^a^	416 ± 33 ^a^
Pectins (mg/L)	562 ± 34 ^a^	640 ± 43 ^a^	780 ± 75 ^b^
Water-soluble pectins (mg/L)	278 ± 37 ^a^	292 ± 65 ^a^	473 ± 81 ^b^
Sucrose (g/L)	18.0 ± 3.9 ^a^	16.8 ± 4.5 ^a^	16.7 ± 5.5 ^a^
Glucose (g/L)	13.1 ± 1.5 ^a^	12.6 ± 2.2 ^a^	9.0 ± 2.5 ^a^
Fructose (g/L)	12.6 ± 1.9 ^a^	12.2 ± 1.9 ^a^	9.7 ± 3.8 ^a^

Means in a row without a common superscript letter differ (*p* < 0.05) as analyzed by two-way ANOVA and the TUKEY test. * as citric acid monohydrate.

**Table 2:** Concentration (mean ± standard deviation, *n* = 3) of flavanone glycosides, quaternary ammonium compounds, and limonoid aglycones in bergamot juice obtained using 3 different juice extraction methods: hand squeezing (Ep-1); FMC (Ep-2); screw press of peeled fruits (Ep-3), and in debittered bergamot juice (dBJ).

	Ep-1	Ep-2	Ep-3	dBJ
*flavanone glycosides*				
neoeriocitrin (mg/L)	114 ± 20 ^a^	219 ± 64 ^b^	402 ± 122 ^c^	7 ± 2 ^d^
narirutin (mg/L)	18 ± 3.0 ^a^	18 ± 3.5 ^a^	37 ± 3.9 ^b^	<0.1
naringin (mg/L)	97 ± 13 ^a^	175 ± 58 ^b^	394 ± 83 ^c^	5 ± 1 ^d^
neohesperidin (mg/L)	66 ± 15 ^a^	124 ± 69 ^b^	279 ± 78 ^c^	4 ± 2 ^d^
*Quaternary ammonium compounds*				
*N*,*N*-dimethyl-*L*-proline (mg/L)	436 ± 49 ^a^	569 ± 126 ^a^	667 ± 147^a^	395 ± 38 ^a^
*limonoid aglycones*				
Limonin (mg/L)	20 ± 4 ^a^	37 ± 13 ^a^	65 ± 14 ^b^	5 ± 1 ^c^
Nomilin (mg/L)	17 ± 4 ^a^	42 ± 11 ^b^	67 ± 12 ^b^	3 ± 1 ^d^

Means in a row without a common superscript letter differ (*p* < 0.05) as analyzed by two-way ANOVA and the TUKEY test.

## References

[1] Codoñer-Franch P., Valls-Bellés V. (2010). Citrus as functional foods. Curr. Top. Nutraceutical Res..

[2] Peterson J., Dwyer J. (1998). Flavonoids: Dietary occurrence and biochemical activity. Nutr. Res..

[3] Benavente-García O., Castillo J. (2008). Update on uses and properties of citrus flavonoids: New findings in anticancer, cardiovascular, and anti-inflammatory activity. J. Agric. Food Chem..

[4] Manners G.D. (2007). Citrus limonoids: Analysis, bioactivity, and biomedical prospects. J. Agric. Food Chem..

[5] Servillo L., Giovane A., Balestrieri M.L., Bata-Csere A., Cautela D., Castaldo D. (2011). Betaines in fruits of citrus genus plants. J. Agric. Food Chem..

[6] Li C., Schluesener H. (2017). Health-promoting effects of the citrus flavanone hesperidin. Crit. Rev. Food Sci. Nutr..

[7] Kim J., Jayaprakasha G.K., Patil B.S. (2013). Limonoids and their anti-proliferative and anti-aromatase properties in human breast cancer cells. Food Funct..

[8] Yu J., Wang L., Walzem R.L., Miller E.G., Pike L.M., Patil B.S. (2005). Antioxidant activity of citrus limonoids, flavonoids, and coumarins. J. Agric. Food Chem..

[9] Yin J., Zhang Z.-W., Yu W.-J., Liao J.-Y., Luo X.-G., Shen Y.-J. (2010). Stachydrine, a Major Constituent of the Chinese Herb *Leonurus heterophyllus* Sweet, Ameliorates Human Umbilical Vein Endothelial Cells Injury Induced by Anoxia-Reoxygenation. Am. J. Chin. Med..

[10] Servillo L., D’Onofrio N., Longobardi L., Sirangelo I., Giovane A., Cautela D., Castaldo D., Giordano A., Balestrieri M.L. (2013). Stachydrine ameliorates high-glucose induced endothelial cell senescence and SIRT1 downregulation. J. Cell. Biochem..

[11] Toth P.P., Patti A.M., Nikolic D., Giglio R.V., Castellino G., Biancucci T., Geraci F., David S., Montalto G., Rizvi A. (2016). Bergamot reduces plasma lipids, atherogenic small dense LDL, and subclinical atherosclerosis in subjects with moderate hypercholesterolemia: A 6 months prospective study. Front. Pharmacol..

[12] Mannucci C., Navarra M., Calapai F., Squeri R., Gangemi S., Calapai G. (2017). Clinical Pharmacology of *Citrus bergamia*: A Systematic Review. Phyther. Res..

[13] Khan I.A., Abourashed E.A. (2009). Leung's Encyclopedia of Common Natural Ingredients Used in Food, Drugs, and Cosmetics.

[14] Cautela D., Laratta B., Santelli F., Trifirò A., Servillo L., Castaldo D. (2008). Estimating bergamot juice adulteration of lemon juice by High-Performance Liquid Chromatography (HPLC) analysis of flavanone glycosides. J. Agric. Food Chem..

[15] Gattuso G., Caristi C., Gargiulli C., Bellocco E., Toscano G., Leuzzi U. (2006). Flavonoid glycosides in bergamot juice (*Citrus bergamia Risso*). J. Agric. Food Chem..

[16] Russo M., Arigò A., Calabrò M.L., Farnetti S., Mondello L., Dugo P. (2016). Bergamot (*Citrus bergamia Risso*) as a source of nutraceuticals: Limonoids and flavonoids. J. Funct. Foods.

[17] Cautela D., Castaldo D., Servillo L., Giovane A., Bayindirli A. (2010). Enzymes in citrus juice processing. Enzymes in Fruit and Vegetable Processing: Chemistry and Engineering Applications.

[18] Tomas-Barberan F.A., Clifford M.N. (2000). Flavanones, chalcones and dihydrochalcones. Nature, occurrence and dietary burden. J. Sci. Food Agric..

[19] Gouws C.A., Georgouopoulou E., Mellor D.D., Naumovski N. (2019). The effect of juicing methods on the phytochemical and antioxidant characteristics of the purple prickly pear (*Opuntia ficus indica*)—Preliminary Findings on Juice and Pomace. Beverages.

[20] Gil-Izquierdo A., Gil M.I., Ferreres F. (2002). Effect of processing techniques at industrial scale on orange juice antioxidant and beneficial health compounds. J. Agric. Food Chem..

[21] Calvarano M., Postorino E., Gionfriddo F., Calvarano I. (1995). Sulla deamarizzazione del succo di bergamotto. Essenz. Deriv. Agrum..

[22] Methods of Analysis (2001). International Federation of Fruit Juice Producers (IFU).

[23] Vella F.M., Cautela D., Laratta B. (2019). Characterization of Polyphenolic Compounds in Cantaloupe Melon By-Products. Foods.

[24] Servillo L., Giovane A., Balestrieri M.L., Cautela D., Castaldo D. (2011). Proline derivatives in fruits of bergamot (*Citrus bergamia Risso* et Poit): Presence of *N*-methyl-l-proline and 4-hydroxy-l-proline betaine. J. Agric. Food Chem..

[25] Esposito C., Tridente R., Balestrieri M.L., Laratta B., Servillo L., Castaldo D. (2006). Distribuzione dei limonoidi nelle diverse parti del frutto di bergamotto. Ess. Deriv. Agric..

[26] Blois M.S. (1958). Antioxidant determinations by the use of a stable free radical. Nature.

[27] Scherer R., Godoy H.T. (2009). Antioxidant activity index (AAI) by the 2,2-diphenyl-1-picrylhydrazyl method. Food Chem..

[28] Horowitz R.M., Cody V., Middleton E., Harborne J., Alan R. (1986). Taste effects of flavonoids. Plant Flavonoids in Biology and Medicine, Biochemical, Pharmacological, and Structure-Activity.

[29] McIntosh C.A., Mansell R.L. (1997). Three-dimensional distribution of limonin, limonoate A-ring monolactone, and naringin in the fruit tissues of three varieties of *Citrus paradisi*. J. Agric. Food Chem..

[30] Croak S., Corredig M. (2006). The role of pectin in orange juice stabilization: Effect of pectin methylesterase and pectinase activity on the size of cloud particles. Food Hydrocoll..

[31] Lloyd A.J., Beckmann M., Favé G., Mathers J.C., Draper J. (2011). Proline betaine and its biotransformation products in fasting urine samples are potential biomarkers of habitual citrus fruit consumption. Br. J. Nutr..

[32] Rangel-Huerta O.D., Aguilera C.M., Perez-de-la-Cruz A., Vallejo F., Tomas-Barberan F., Gil A., Mesa M.D. (2017). A serum metabolomics-driven approach predicts orange juice consumption and its impact on oxidative stress and inflammation in subjects from the BIONAOS study. Mol. Nutr. Food Res..

[33] Jung U.J., Kim H.J., Lee J.S., Lee M.K., Kim H.O., Park E.J., Kim H.K., Jeong T.S., Choi M.S. (2003). Naringin supplementation lowers plasma lipids and enhances erythrocyte antioxidant enzyme activities in hypercholesterolemic subjects. Clin. Nutr..

[34] Kaur J., Kaur G. (2015). An insight into the role of citrus bioactives in modulation of colon cancer. J. Funct. Foods.

[35] Kimball D.A., Norman S.I. (1990). Processing Effects during Commercial Debittering of California Navel Orange Juice. J. Agric. Food Chem..

[36] Miller N.J., Rice-Evans C.A. (1997). The relative contributions of ascorbic acid and phenolic antioxidants to the total antioxidant activity of orange and apple fruit juices and blackcurrant drink. Food Chem..

